# Resource Use and Cost Associated With Cardiovascular, Renal, Bone, and Neuropsychiatric Comorbidities in People With HIV in Spain

**DOI:** 10.36469/001c.144019

**Published:** 2025-10-20

**Authors:** Pere Ventayol, Carlos Dueñas, Carlos Martín, Antonio Castro, Belén Citoler, Neus Vidal-Vilar

**Affiliations:** 1 Farmacia Hospitalaria Hospital Universitario Son Espases, Palmas, Spain; 2 Medicina Interna Hospital Clínico Universitario de Valladolid, Valladolid, Spain; 3 Medicina Interna Complejo Hospitalario de Cáceres, Cáceres, Spain; 4 Gilead Sciences Inc., Madrid, Spain; 5 Outcomes’10 S.L., (a ProductLife Group Company), Castellón de la Plana, Spain

**Keywords:** HIV, comorbidities, healthcare cost, antiretroviral therapy toxicity, economic impact, cost analysis

## Abstract

**Background:** Antiretroviral therapies (ART) have significantly improved the life expectancy of people with HIV (PLWH). However, chronic immune activation and some ART regimens may increase the prevalence of non-HIV comorbidities, such as cardiovascular, renal, bone, and neuropsychiatric conditions. These comorbidities increase healthcare resource utilization and costs for the Spanish National Health System (NHS), yet data on their economic impact remain scarce. **Objective:** To estimate the healthcare resource use and costs associated with cardiovascular, renal, bone, and neuropsychiatric comorbidities in PLWH from the Spanish NHS perspective and to simulate the financial impact of a potential prevalence increase due to ART toxicity. **Methods:** An Excel-based model was used to compare a current scenario using national epidemiological data and an alternative scenario with increased comorbidity prevalence due to ART toxicity. Two cohorts were analyzed: PLWH diagnosed for less than 10 years and those diagnosed for 10 years or more. Epidemiological and healthcare utilization data were collected from the literature and validated by an expert panel. Direct healthcare costs, including hospitalizations, tests, medical visits, and emergency care, were estimated and discounted at a 3% annual discount rate. **Results:** In 2024, 139 390 PLWH would be living in Spain, with 17 046 having cardiovascular, 7752 renal, 17 700 bone, and 16 207 neuropsychiatric comorbidities, predominantly affecting patients diagnosed for at least 10 years. By 2034, these figures will rise to 33 555, 15 391, 33 950, and 27 388, respectively, with increases observed in both cohorts. Estimated 2024 healthcare costs were €83 million, €48 million, €55 million, and €97 million for cardiovascular, renal, bone, and neuropsychiatric comorbidities, respectively. The alternative scenario with increased comorbidities prevalence projected an additional €900 million to €1400 million. **Discussion:** The projected increase in the prevalence of cardiovascular, renal, bone, and neuropsychiatric comorbidities among PLWH represents a significant challenge for the Spanish NHS, primarily driven by long-term use of specific ART regimes associated with higher toxicity profiles. **Conclusion:** Non-HIV comorbidities pose a growing economic challenge. Selecting lower-toxicity ART regimens and preventive strategies will be crucial to mitigating financial impact.

## INTRODUCTION

The development of antiretroviral therapies (ART) has been a breakthrough in the treatment and management of people with HIV (PLWH). In the past decades, the use of ART has delayed or suppressed AIDS progression,^1,2^ bringing the life expectancy of PLWH close to that of individuals without HIV.^3,4^ As a result, an increase in older people living with HIV is expected in the following years, with the proportion of patients 50 years or older rising to 73% in 2030.^5^

Although ART improves the lives of PLWH, the chronic immune system activation that occurs in these individuals might increase the number of non-HIV comorbidities such as cardiovascular disease, kidney disease, and decreased bone mineral density,^6–8^ especially in those over 55 years old.^9^ Moreover, the use of certain ART regimens might contribute to increased rates of these comorbidities and the emergence of neuropsychiatric symptoms. Previous studies have reported associations between tenofovir disoproxil and renal or bone effects, boosted protease inhibitors (eg, darunavir) and adverse lipid or cardiovascular profiles, and integrase inhibitors (eg, dolutegravir, bictegravir) and neuropsychiatric symptoms.^7,10–12^ In line with recent evidence, adults with HIV show a higher multimorbidity and comedication burden that matched individuals without HIV, with cardiovascular, renal and mental health conditions among the most frequent, underscoring the need to consider comorbidities and comedications when choosing ART.^13,14^

In addition to worsening the health status of PLWH, non-HIV comorbidities are associated with a high use of healthcare resources and costs. Studies conducted in different settings reported that PLWH with cardiovascular disease, chronic kidney disease, and fracture/osteoporosis showed higher use of resources than matched controls, resulting in greater costs.^15–17^ However, data from Spanish settings are scarce.

In this context, the aims of the study were to (1) estimate the healthcare resource use and costs associated with cardiovascular, renal, bone, and neuropsychiatric comorbidities in PLWH in Spain from the perspective of the Spanish National Health System (NHS), and (2) simulate the budgetary impact of plausible increases in their prevalence potentially related to ART toxicity for regimens commonly used in Spain (darunavir, dolutegravir, bictegravir, and tenofovir disoproxil).

## METHODS

### Study Design

An Excel-based deterministic cohort model (ie, a static-cohort epidemiologic model with 1-year cycles) was developed to compare two scenarios: a current scenario based on existing national epidemiological data and an alternative scenario where the prevalence of considered comorbidities increases due to HIV treatment-related toxicity (see **Supplementary Figure S1**). Direct healthcare costs associated with resource use for PLWH with cardiovascular, renal, bone and neuropsychiatric comorbidities were estimated for the period 2024-2034.

In each scenario, two cohorts were considered: patients diagnosed with HIV less than 10 years ago (diagnosis <10 years) and those diagnosed at least 10 years ago (diagnosis ≥10 years), capturing differences in age and cumulative ART exposure. The <10 and ≥10 years since-diagnosis thresholds were chosen because duration of HIV infection is a key driver of comorbidity risk, reflecting aging and cumulative ART exposure.^9^ In addition, outcomes were stratified by age <50 or ≥50 years, consistent with definitions of older PLWH in the literature.^8^

Model inputs derived from published literature and national statistics were reviewed for clinical plausibility by a panel of four HIV clinicians (hospital pharmacists, internists, and infectious disease specialists) from different Spanish hospitals. The experts, all with extensive experience in HIV patient management, assessed whether the epidemiological, clinical, and cost assumptions used in the model were consistent with current Spanish clinical practice.

### Epidemiological Data

The number of PLWH was estimated based on the total population of Spain in 2024 according to the National Statistics Institute^18^ and an estimated HIV prevalence of 0.31%.^19^ Of those individuals, 92.5% were considered diagnosed, of which 39.6% had been diagnosed <10 years, based on national data.^19^ In addition, an annual mortality rate of 20.7 per 1000 persons with HIV was applied.^3^

To estimate the projection of PLWH with each comorbidity from 2024 to 2034, the baseline prevalence (**Supplementary Table S1**) and annual incidence (**Supplementary Table S2**) of each comorbidity per cohort in 2024 were obtained from the literature.^20^

Additionally, annual incidence data were differentiated according to the mean age of each cohort, distinguishing between patients >50 and those ≤50 years. Mean ages of 34 and 48 years were considered for cohorts with a diagnosis of <10 and ≥10 years,^9^ respectively. To maintain a conservative approach, a 1% annual reduction in the incidence of comorbidities was assumed to reflect the improvements in current treatments and the reduction in toxicity that these improvements imply.

### Cost Analysis

The analysis considered direct health costs (in 2024 euros). Hospital admissions, diagnostic tests, emergency department visits, outpatient visits, and laboratory tests were considered. **Table 1** shows the resource use per comorbidity obtained from the literature^20^ and its unit cost obtained from the Spanish health care cost database eSalud.^21^ All costs were inflated from their origin year to 2024 using the consumer price index published by the Spanish Statistical Office.^22^ An annual discount rate of 3% was applied.^23^

**Table 1.** Cost and Resource Use per Comorbidity and Year

**Resource**	**Cost, €**	**Cardiovascular**	**Renal**	**Bone**	**Neuropsychiatric**
Hospital admissions	4669.00	0.63	0.84	0.32	0.83
Diagnostic tests	157.23	3.88	4.60	3.23	4.24
ED visits	225.93	1.31	1.98	0.80	1.95
Non-HIV visits (outpatient)	158.21	1.62	1.72	1.30	1.67
HIV visits	158.21	3.21	3.06	2.90	3.02
Blood tests	30.96	9.20	8.64	7.85	8.43

Abbreviation: ED, emergency department.

### Alternative Scenario

The alternative scenario was configured based on the available treatments, using prevalence increase estimates derived from the expert committee’s opinion, which was complemented by the most recent literature^11^ due to the limited information available for the initial estimation. To further illustrate the prevalence scenarios of cardiovascular and neuropsychiatric comorbidities, the most commonly used treatments in Spain, as reported in the 2024 hospital survey of patients with HIV/AIDS,^19^ were selected (darunavir, 15%; dolutegravir, 37.1%; and bictegravir, 31.7%). The multipliers (**Table 2**) were applied as full-exposure counterfactuals (not market share–weighted) to isolate regimen effects. Although tenofovir disoproxil is less frequently prescribed today, having been largely replaced by tenofovir alafenamide since its introduction in 2017, it was included in the analysis due to its well-documented renal and bone-related risks,^12,24,25^ which remain relevant for comparative purposes. **Table 2** values represent absolute percentage-point increases to the 2024 baseline prevalence for each comorbidity.

**Table 2.** Increased Baseline Prevalence Percentage

**Comorbidity**	**Years Since Diagnosis**		**Source**
	**<10**	**≥10**	
Cardiovascular (darunavir)	1.13%	1.88%	Expert opinion
Renal (tenofovir disoproxil)	2.93%	7.23%	Expert opinion
Bone (tenofovir disoproxil)	2.38%	4.66%	Expert opinion
Neuropsychiatric			
Dolutegravir	1.88%	2.05%	Expert opinion
Bictegravir	1.25%	2.00%	Expert opinion
Dolutegravir	2.58%		Pérez-Valero^11a^
Bictegravir	0.70%		Pérez-Valero^11a^

^a^The p_50_ estimate is used for both cohorts since the estimates do not vary according to the time of diagnosis.

The increases in the baseline prevalence of comorbidities were used to calculate how costs increased in both cohorts during the 2024–2034 period (**Table 2**). The incidence figures remained the same as in the current scenario, because this scenario is designed to capture an increased prevalent burden at model entry attributable to prior ART exposure; holding incidence constant isolates this effect and avoids compounding in the absence of robust regimen-specific incidence multipliers. Finally, the results of the alternative scenarios were compared with the costs of the current scenario.

### Sensitivity Analysis

Two sensitivity analyses (SA) were performed to show the variation in the results due to a change in the prevalence of comorbidities. Prevalence from different national studies was considered. The first SA used comorbidity prevalence and age data from the Cohort of the Spanish HIV/AIDS Research Network (CoRIS) study for the cohort diagnosed <10 years ago.^26^ The second SA used the prevalence of comorbidities from the VACH study for the cohort with a diagnosis ≥10 years ago.^9^ The prevalence considered for each SA is shown in **Supplementary Table S3**.

## RESULTS

A total of 139 390 PLWH were estimated in 2024 in Spain (55 199 patients with a diagnosis <10 years ago and 84 192 patients with a diagnosis ≥10 years ago). At the end of the time horizon, in 2034, 113 081 patients remained in the model (44 780 patients with a diagnosis <10 years ago and 68 301 patients with a diagnosis ≥10 years ago).

### Current Scenario

The estimated number of PLWH with cardiovascular, renal, bone, and neuropsychiatric comorbidities in the current scenario in 2024 was 17 046, 7752, 17 700 and 16 207, respectively. At the end of the time horizon (2034), it was estimated that 33 555, 15 391, 33 950, and 27 388 PLWH would have the comorbidities above, respectively. An increase in PLWH with comorbidities over time was observed, being more common in patients diagnosed ≥10 years (**Table 3**).

**Table 3.** Number of People Living With HIV With Each Comorbidity According to Cohort and Year of Analysis

**Comorbidity**	**<10 Years Since Diagnosis**			**≥10 Years Since Diagnosis**			**Total^a^ (PY = 1 384 058)**
**2024 (n = 55 199)**	**2034 (n = 44 780)**	**Total^a^ (PY = 548 087)**	**2024 (n = 84 192)**	**2034 (n = 68 301)**	**Total^a^ (PY = 835 971)**		
Cardiovascular	2650	5166	44 468	14 397	28 389	242 533	287 002
Renal	1270	2583	21 903	6483	12 808	108 575	130 479
Bone	3809	8241	69 106	13 892	25 709	224 191	293 297
Neuropsychiatric	2484	5606	46 333	13 723	21 782	201 075	247 407

Abbreviation: PY, person-years.

Values are number of patients with the comorbidity during the study period.

^a^Corresponds to the sum of annual patients during the study period (2024-2034).

**Figure 1.**
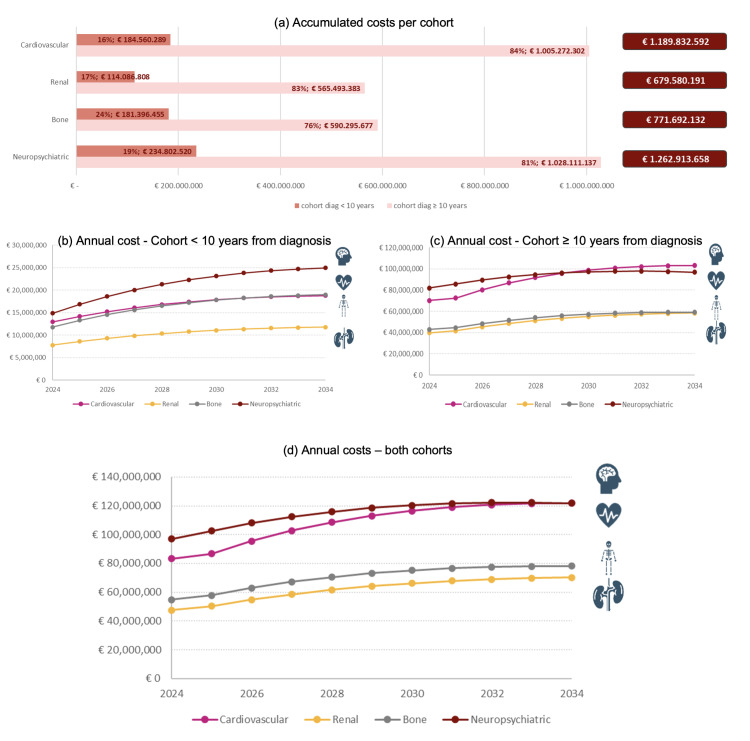
Total Costs: 2024-2034

In 2024, PLWH with cardiovascular, renal, bone, and neuropsychiatric comorbidities accounted for €83.19 million, €47.53 million, €54.77 million, and €96.92 million, respectively (**Supplementary Table S4**). Neuropsychiatric comorbidities were associated with the highest costs. A total of €1190 million, €680 million, €772 million, and €1263 million were accumulated for each comorbidity during the whole period (**Figure 1a**). Of these total costs, 84%, 83%, 76%, and 81% corresponded to the cohort with a diagnosis ≥10 years ago.

Comorbidity costs increase over the years and tend to stabilize, with similar behavior in both cohorts (**Figure 1b and 1c**). In the cohort with a diagnosis ≥10 years ago, a slight increase was observed in 2026 due to the simulated cohort reaching an age of 50 years old, which affects the incidence of comorbidities. It is worth noting that costs increase even though the number of PLWH in the model follows a decreasing trend.

### Alternative Scenarios

A total of 306 399, 203 252, 339 697, 280 573, and 256 406 person-years with cardiovascular (darunavir), renal (tenofovir), bone (tenofovir), and neuropsychiatric (dolutegravir and bictegravir) comorbidities, respectively, were estimated in the alternative scenarios using prevalence increases based on expert opinions. Additionally, 272 839 and 269 172 person-years with neuropsychiatric comorbidities (dolutegravir and bictegravir, respectively) were estimated based on prevalence increases obtained from literature (**Table 4**). These new figures imply an increase of between 4% and 56% in person-years of comorbidities compared with the current scenario.

**Table 4.** Alternative Scenario Results (Person-Years)

	**<10 Years Since Diagnosis**			**≥10 Years Since Diagnosis**			**Total^a^ (PY = 1 384 058)**
**2024 (n = 55 199)**	**2034 (n = 44 780)**	**Total^a^ (PY = 548 087)**	**2024 (n = 84 192)**	**2034 (n = 68 301)**	**Total^a^ (PY = 835 971)**		
Cardiovascular (darunavir)^b^	3271	5635	50 426	15 975	29 295	255 973	306 399
Renal (tenofovir disoproxil)^b^	2884	3849	37 673	12 566	17 163	165 579	203 252
Bone (tenofovir disoproxil)^b^	5120	9175	81 338	17 813	28 092	258 359	339 697
Neuropsychiatric							
Dolutegravir^b^	3519	6377	56 195	15 449	22 925	216 644	272 839
Bictegravir^b^	3174	6120	52 908	15 407	22 897	216 264	269 172
Dolutegravir^c^	3908	6667	59 903	15 895	23 220	220 670	280 573
Bictegravir^c^	2870	5894	50 015	14 313	22 172	206 391	256 406

Abbreviation: PY, person-years.

^a^Corresponds to the sum of annual patients during the study period (2024-2034).

^b^Expert source of the prevalence increase.

^c^Literature source of the prevalence increase.

**Figure 2.**
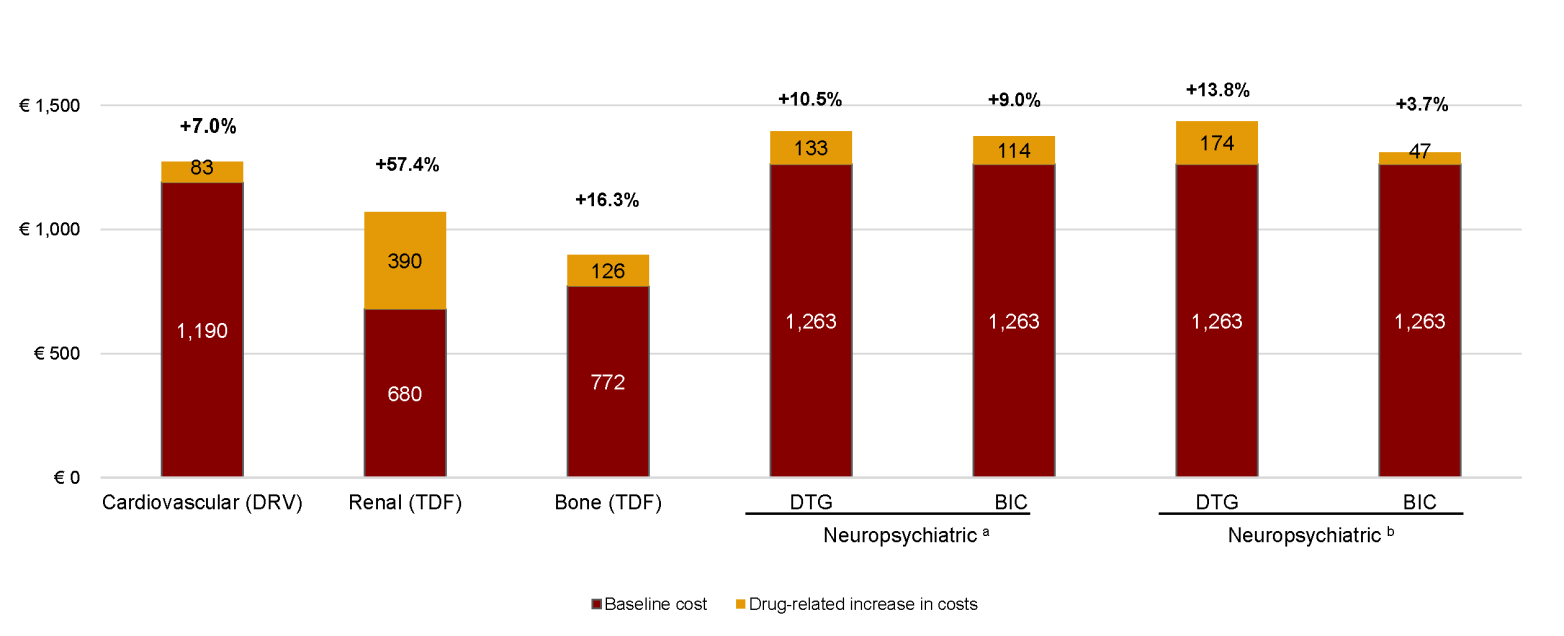
Alternative Scenario Result: Costs 2024-2034 (in Million €) Abbreviations: BIC, bictegravir; DRV, darunavir; DTG, dolutegravir; e, expert source of prevalence increase; literature source of prevalence increase; TDF, tenofovir disoproxil.

An increase in the prevalence of comorbidity meant an increase in the use of healthcare resources and, therefore, health care costs. The estimated cost increase would lead to a financial burden of €900 to €1400 million in the entire 2024-2034 period as shown in **Figure 2**.

### Sensitivity Analysis

In both the first and second sensitivity analyses, fewer people with comorbidities were estimated (**Supplementary Table S5**).

In the first sensitivity analysis, using CoRIS prevalence and age for the cohort with a diagnosis <10 years ago, a difference of €62 020 247, €9 135 439, and €28 052 159 was obtained with respect to the base case over the entire period for cardiovascular, renal, and neuropsychiatric comorbidities, respectively (**Supplementary Figure S2**) (the CoRIS study did not include bone comorbidities).

Modifying the prevalence in the cohort with a diagnosis ≥10 years with a second SA using the VACH^9^ study resulted in a difference of €382 080 142, €74 217 924, and €107 772 536 for cardiovascular, renal, and bone respectively (**Supplementary Figure S2**) (neuropsychiatric comorbidities were not included in this study).

## DISCUSSION

This study estimated the costs associated with cardiovascular, renal, bone, and neuropsychiatric comorbidities in PLWH over 10 years from 2024 to 2034. Our analysis demonstrates that even a modest increase the prevalence of these comorbidities, potentially due to the toxicity of specific ART regimens, can lead to substantial long-term economic impacts on the Spanish NHS. Specifically, we projected that the number of PLWH with cardiovascular comorbidities would nearly double from 17 046 in 2024 to 33 555 in 2034. Similar upward trends were observed for renal, bone, and neuropsychiatric comorbidities, with the number of patients affected by bone comorbidities, increasing from 17 700 to 33 950, and those with neuropsychiatric issues, rising from 16 207 to 27 388, over the same period. These increases were more pronounced among patients diagnosed with HIV ≥10 years ago, highlighting the cumulative effect of long-term HIV infection and treatment. As can be observed, despite the constant decrease in PLWH throughout the entire study period, the costs attributable to comorbidities keep increasing due to their incidence.

The increasing prevalence of these comorbidities leads to substantial growth in healthcare resource utilization and associated costs. Neuropsychiatric comorbidities, in particular, incurred the highest annual expenses among the conditions studied, reaching €96.92 million in 2024 and accumulating to more than €1260 million by 2034 in the current scenario. In the alternative scenario, which incorporates higher prevalence rates, the prevalence increases applied to dolutegravir resulted in higher projected costs than those applied to bictegravir, based on both expert opinions (€1.396 billion vs €1.376 billion) and the literature (€1.437 billion vs €1.310 billion). Thus, compared with the current scenario, dolutegravir would result in an incremental cost of 10% to 14%, while the incremental cost for bictegravir would range between 4% and 9%.

Significant cost increases for comorbidities were also observed with tenofovir disoproxil, specifically, a 55.77% rise in renal comorbidity costs and a 15.2% increase in bone-related costs compared with the current scenario. Similarly, under the assumptions of the alternative scenario, applying the prevalence increase linked to darunavir use led to a projected 6.76% increase in cardiovascular comorbidity costs relative to the current scenario.

Previous studies have shown that the presence of non-HIV comorbidities in PLWH results in high costs for the NHS and for the PLWH themselves. For example, Christensen et al^27^ conducted a retrospective claims analysis in Germany and found that PLWH incurred mean total costs (excluding ART) of €8049, compared with €3658 in a matched non-HIV cohort. This difference was primarily attributed to the higher prevalence of comorbidities such as cardiovascular disease and chronic renal disease in the HIV population. Additionally, they reported that PLWH had significantly higher outpatient and inpatient costs, with excess costs of €1441 and €321, respectively. Similarly, Van Duin et al^28^ assessed HIV patients in Colombia and found that although the total costs were not significantly higher for HIV patients with comorbidities compared with those without, patients with multiple comorbidities had significantly lower utility scores, indicating a reduced quality of life.

Other studies have evaluated the healthcare costs related to non-HIV comorbidities over time. Hjalte et al reported a higher mean excess cost due to cardiovascular disease, chronic kidney disease, and osteoporotic fractures attributable to HIV compared with individuals without HIV, especially for PLWH >50 years old.^29^ In this study, chronic kidney disease was the most significant contributor to the excess cost of these comorbidities, while in our study, chronic kidney disease was the comorbidity with the lowest excess cost in all analyses. However, the conclusions of this study may not be comparable since not all neuropsychiatric or cardiovascular comorbidities are considered, and the treatment regimens used may be different. Ehlers et al^30^ showed that a cohort of PLWH had higher costs in the 3 years prior to diagnosis and in the first 9 years after diagnosis compared with a non-HIV cohort. When costs related to HIV treatment were excluded, PLWH incurred costs twice as high as those in the non-HIV cohort. In addition, the authors reported that the cost of treating non-HIV comorbidities in PLWH during the study period was higher than the cost of treating HIV.^30^

Considering our results, those published previously, the fact that the comorbidities evaluated in the present study are highly prevalent among PLWH compared with individuals without HIV,^27^ and that it translates into a high cost for the Spanish NHS, screening for these comorbidities and selecting the most appropriate ART would result in reduced HRU and costs.

Given that neuropsychiatric comorbidities were the largest driver of healthcare resource utilization and costs in our projections, systematic detection and early management should be a priority in HIV care. In line with the CONECTAR Project recommendations,^31^ screening for anxiety, depression, and insomnia should be implemented at baseline and repeated annually or biannually depending on patient risk, using validated tools such as the Generalized Anxiety Disorder 2-Item (GAD-2), the Hospital Anxiety and Depression Scale (HADS), or the Pittsburgh Sleep Quality Index (PSQI). At-risk populations (such as those with a history of psychiatric disorders, substance use, cognitive decline, multiple comorbidities, or treatment with potentially neurotoxic ART) should be prioritized. When identified, management should follow a stepped approach, starting with nonpharmacological interventions (eg, cognitive behavioral therapy, psychoeducation, sleep hygiene), complemented by pharmacological treatment when clinically indicated, and with clear referral pathways for severe or nonresponsive cases. Training healthcare professionals, as shown by Blanch et al,^31^ improves knowledge, confidence, and use of these strategies, and can help reduce the clinical and economic burden of neuropsychiatric comorbidities in PLWH.

Our study has some limitations. One of the limitations is related with the study methodology. The economic modeling of a pathology allows us to project what might be observed under a series of assumptions but does not allow us to predict the future. In the coming years, the potential development of new therapies may cause the scenarios proposed to differ slightly from those expected in the established time horizon. In addition, these new therapeutic options would be involved in HIV prevention and epidemiological estimates may therefore be altered after the introduction of these treatments through a reduction in the incidence of the disease. However, our projections are based on the best available evidence to date, providing valuable insight into the significance of non-HIV comorbidities when making treatment decisions.

Another limitation is the estimation of the increase in comorbidities in alternative scenarios. The negative effects that certain treatments can cause are well known, but quantifying this variation numerically is a challenge. However, a panel of experts validated all inputs used in the study, and they provided consistent estimates with minimal variability in their independent assessments. This expert input enhances the reliability of our projections despite the inherent difficulty of quantifying such variations.

Additionally, in the alternative scenario, regimen-specific prevalence multipliers were applied as full-exposure counterfactuals rather than weighted by current market shares. This approach was chosen to isolate potential toxicity-related effects and avoid embedding a single, time-specific treatment mix into long-term projections. Finally, the regimen-specific prevalence multipliers in the alternative scenario were partly based on experts’ opinion because robust causal estimates by regimen and age are scarce. Accordingly, this scenario should be interpreted as exploratory and hypothesis-generating; future work should test a broader range of multipliers as data accumulate.

Although our model is parametrized with Spanish epidemiological data, practice patterns, and unit costs, several features support transferability to other high-income health systems. First, the clinical mechanism underpinning our results (the accumulation of non-HIV comorbidities with aging and long-term ART exposure) is common across settings. Second, the ART classes modeled and their known toxicity profiles are common across international guidelines. Third, the relative cost pressures we identified stem from resource-use patterns that are not unique to Spain. Nevertheless, the absolute budget impact should be interpreted cautiously outside Spain because unit prices, service delivery models, and ART market shares differ across countries.

## CONCLUSION

This study estimated the costs associated with cardiovascular, renal, bone, and neuropsychiatric comorbidities in PLWH in Spain over a 10-year period (2024-2034). The findings highlight that even a moderate increase in the prevalence of these comorbidities (potentially due to the toxicity of specific antiretroviral regimens) can have significant impacts on the Spanish National Health System. In this context, during the next years it will be essential to implement strategies aimed at minimizing the increase in comorbidities caused by treatment toxicity in PLWH.

### Disclosures

A.C. is an employee at Gilead Sciences SLU. B.C. and N.V. are employees at Outcomes’10 (a ProductLife Group Company).

## Supplementary Material

Online Supplementary Material
