# Supplementary material for: Resource Use and Cost Associated With Cardiovascular, Renal, Bone, and Neuropsychiatric Comorbidities in People With HIV in Spain
Source: J Health Econ Outcomes Res. 2025 Oct 20;12(2):147–53. doi: 10.36469/001c.144019 (PMC12542903; doi:10.36469/001c.144019)
Supplement: Online Supplementary Material [file jheor_2025_12_2_144019_306966.pdf]

Journal of Health Economics and Outcomes Research

## Online Supplementary Material

Resource use and cost associated with cardiovascular, renal, bone and neuropsychiatric comorbidities in people with HIV in Spain. *JHEOR*. 2025;12(2):147-153. doi:10.36469/jheor.2025.143019

**Figure S1: Model Schema**

**Table S1: Baseline Prevalence of Comorbidities**

**Table S2: Annual Incidence of Comorbidities (2024-2034)**

**Table S3: Sensitivity Analysis Parameters**

**Table S4: Cost per Year in the Current Scenario (Million Euros)**

**Table S5: Sensitivity Analysis Results (Person-Years)**

**Figure S2: Sensitivity Analysis Results (Costs)**

This supplementary material has been provided by the authors to give readers additional information about their work.

This is an open-access article distributed under the terms of the Creative Commons Attribution 4.0 International License (CCBY-4.0). View this license's legal deed at http://creativecommons.org/licenses/by/4.0 and legal code at http://creativecommons.org/licenses/by/4.0/legalcode for more information.

**Figure S1.** Model Schema

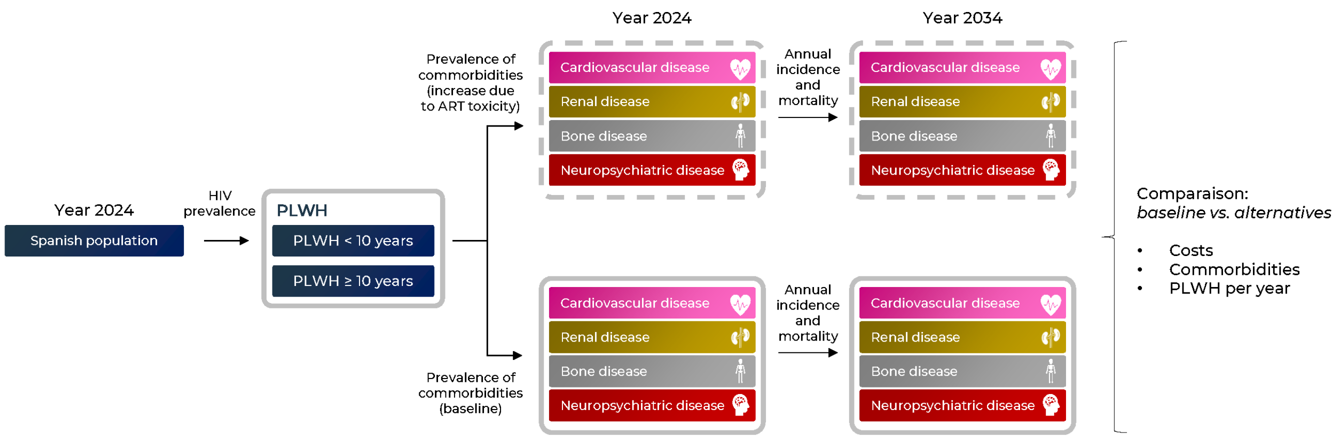


**Table S1.** Baseline Prevalence of Comorbidities

| Comorbidity | Prevalence Cohort <10 Years from Diagnosis (%) | Prevalence Cohort ≥10 Years from Diagnosis (%) |
|---|---|---|
| Cardiovascular | 4.80 | 17.10 |
| Renal | 2.30 | 7.70 |
| Bone | 6.90 | 16.50 |
| Neuropsychiatric | 4.50 | 16.30 |

**Table S2.** Annual Incidence of Comorbidities (2024-2034)

| Comorbidity | Incidence <10 Years from Diagnosis (%) | | Incidence ≥10 Years from Diagnosis (%) | |
|---|---|---|---|---|
| | <50 y | ≥ 50 y | <50 y | ≥ 50 y |
| Cardiovascular | 0.77 | 3.40 | 1.80 | 3.83 |
| Renal | 0.38 | 1.36 | 0.86 | 1.39 |
| Bone | 1.38 | 1.59 | 1.82 | 3.17 |
| Neuropsychiatric | 0.92 | 0.68 | 1.95 | 2.17 |

**Table S3.** Sensitivity Analysis Parameters

| | Prevalence (%) | |
|---|---|---|
| | SA 1: Cohort With Diagnosis <10 y | SA 2: Cohort With Diagnosis ≥10 y |
| Cardiovascular | 2.05 | 4.70 |
| Renal | 1.98 | 5.95 |
| Bone | – | 11.10 |
| Neuropsychiatric | 3.48 | – |

Abbreviation: SA, sensitivity analysis.

**Table S4.** Cost per Year in the Current Scenario

| | Year | Cost in Million Euros (€) | | |
|---|---|---|---|---|
| | | **Diagnosis < 10 y Ago** | **Diagnosis ≥ 10 y Ago** | **Total** |
| Cardiovascular | 0 | 12.93 | 70.26 | 83.19 |
| | 1 | 14.15 | 72.58 | 86.74 |
| | 2 | 15.20 | 80.38 | 95.58 |
| | 3 | 16.07 | 86.73 | 102.80 |
| | 4 | 16.80 | 91.80 | 108.60 |
| | 5 | 17.40 | 95.75 | 113.14 |
| | 6 | 17.87 | 98.72 | 116.59 |
| | 7 | 18.23 | 100.84 | 119.06 |
| | 8 | 18.49 | 102.21 | 120.70 |
| | 9 | 18.66 | 102.93 | 121.59 |
| | 10 | 18.76 | 103.09 | 121.85 |
| Renal | 0 | 7.78 | 39.75 | 47.53 |
| | 1 | 8.59 | 41.66 | 50.24 |
| | 2 | 9.27 | 45.43 | 54.71 |
| | 3 | 9.86 | 48.61 | 58.47 |
| | 4 | 10.35 | 51.24 | 61.60 |
| | 5 | 10.76 | 53.39 | 64.15 |
| | 6 | 11.09 | 55.10 | 66.19 |
| | 7 | 11.35 | 56.42 | 67.78 |
| | 8 | 11.55 | 57.39 | 68.95 |
| | 9 | 11.69 | 58.05 | 69.75 |
| | 10 | 11.78 | 58.43 | 70.22 |
| Bone | 0 | 11.78 | 42.98 | 54.77 |
| | 1 | 13.27 | 44.60 | 57.87 |
| | 2 | 14.54 | 48.42 | 62.96 |
| | 3 | 15.61 | 51.51 | 67.12 |
| | 4 | 16.50 | 53.98 | 70.48 |
| | 5 | 17.22 | 55.89 | 73.12 |
| | 6 | 17.81 | 57.31 | 75.12 |
| | 7 | 18.26 | 58.30 | 76.56 |
| | 8 | 18.60 | 58.91 | 77.51 |
| | 9 | 18.83 | 59.19 | 78.03 |
| | 10 | 18.97 | 59.19 | 78.16 |
| Neuropsychiatric | 0 | 14.85 | 82.07 | 96.92 |
| | 1 | 16.86 | 85.78 | 102.64 |
| | 2 | 18.58 | 89.52 | 108.10 |
| | 3 | 20.04 | 92.45 | 112.50 |
| | 4 | 21.28 | 94.67 | 115.94 |
| | 5 | 22.30 | 96.25 | 118.55 |
| | 6 | 23.14 | 97.26 | 120.40 |
| | 7 | 23.80 | 97.78 | 121.58 |
| | 8 | 24.32 | 97.86 | 122.18 |
| | 9 | 24.69 | 97.56 | 122.25 |
| | 10 | 24.95 | 96.92 | 121.87 |

**Table S5.** Sensitivity Analysis Results (Person-Years)

| | Current Scenario (PY) | SA 1: CoRIS Prevalence (PY) | SA 1: Difference With Current Scenario* (PY) | SA 2: VACH Prevalence (PY) | SA 2: Difference With Current Scenario* (PY) |
|---|---|---|---|---|---|
| Cardiovascular | 287 002 | 272 453 | -14 549 | 198 113 | -88 889 |
| Renal | 130 479 | 128 771 | -1707 | 116 645 | -13 834 |
| Bone | 293 297 | – | – | 253 682 | -39 614 |
| Neuropsychiatric | 247 407 | 242 040 | -5368 | – | – |

Abbreviations: CoRIS, Cohort of the Spanish HIV/AIDS Research Network; PY, person-years; SA, sensitivity analysis.

**Figure S2.** Sensitivity Analysis Results: Costs

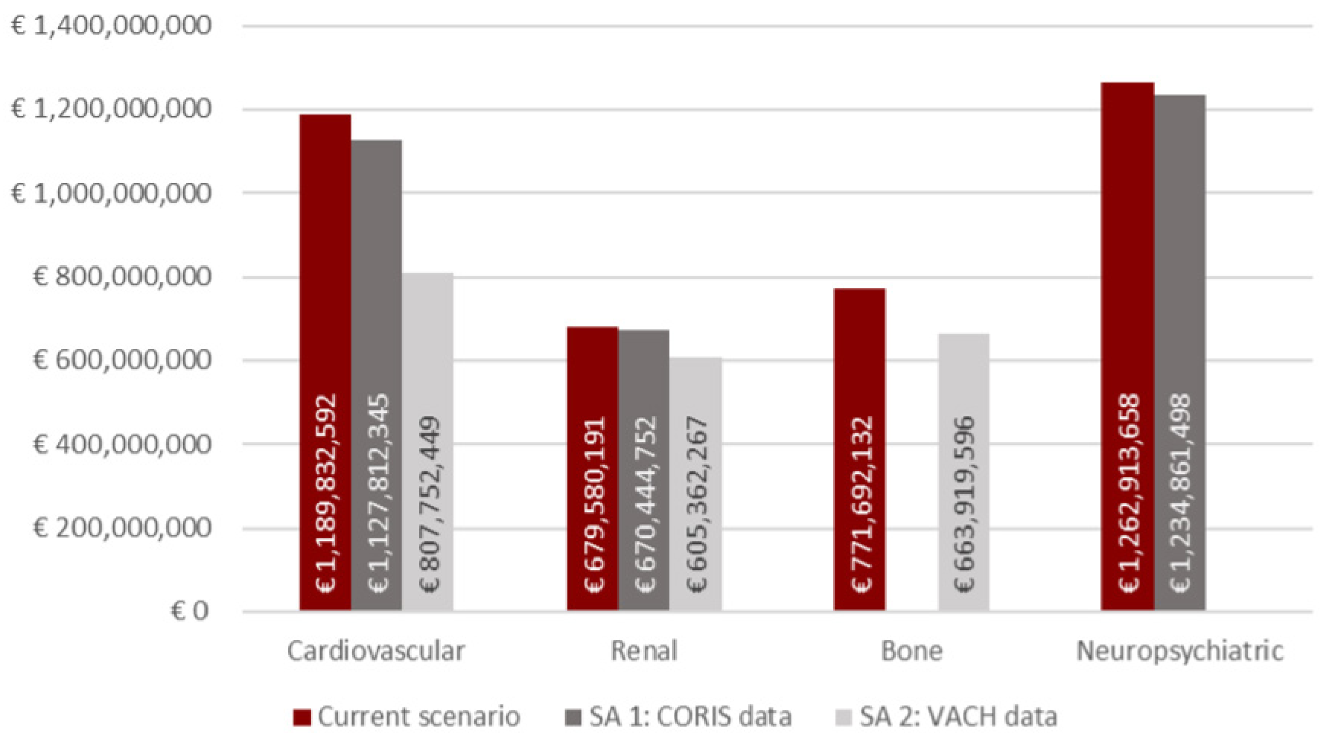


Abbreviations: CoRIS, Cohort of the Spanish HIV/AIDS Research Network; PY, person-years; SA, sensitivity analysis.
